# Risk factors, follow-up, and treatment of urethral recurrence following radical cystectomy and urinary diversion for bladder cancer: a meta-analysis of 9498 patients

**DOI:** 10.18632/oncotarget.23451

**Published:** 2017-12-19

**Authors:** Xinyuan Li, Wuwan Wang, Gongmin Zhu, Weiyang He, Xin Gou

**Affiliations:** ^1^ Department of Urology, The First Affiliated Hospital, Chongqing Medical University, Chongqing, China; ^2^ Department of Cardiology, The First Affiliated Hospital, Chongqing Medical University, Chongqing, China

**Keywords:** urinary bladder neoplasms, cystectomy, ureteral neoplasms, recurrence, risk factors

## Abstract

**Purpose:**

Patients frequently undergo radical cystectomy and urinary diversion for treatment of bladder cancer. However, they remain at risk of urethral recurrence (UR). Studies have determined various risk factors leading to urethral recurrence. However, no publications have weighed the predictive values of these factors.

**Materials and Methods:**

Studies published between 1971 and 2016 were retrieved from PubMed, EMBASE and MEDLINE. We used STATA software (Version 12.0) to estimate the pooled risk ratio.

**Results:**

Twenty-five publications with 9498 patients were included. Overall, male patients, especially those with concomitant carcinoma *in situ*, superficial or intravesical bladder cancer, non-orthotopic diversion, prostatic involvement, bladder neck involvement, positive urethral margins or multifocal bladder cancer were at higher risk of urethral recurrence. The overall risks of recurrence, reported as risk ratios, varied widely. Among all 25 studies, 118 (60.2%) cases in 9 studies were diagnosed through routine follow-up. Another 82 (40.8%) patients in 11 studies first reported symptomatic abnormalities. Prognoses were worse for patients with symptomatic recurrence. Urethral cytology was the most common diagnostic method. Treatment after UR was reported for 272 cases in 14 publications, and 190 patients underwent urethrectomy and 52 underwent urethra-sparing treatments. Outcomes after UR were described in 12 studies reporting 180 cases, and 41 patients were alive through the end of follow-up and 65 patients died of bladder cancer.

**Conclusions:**

UR following radical cystectomy for bladder cancer was closely related to risk factors. Precautions, strict follow-up protocols and rational therapies were critical to patients with high risks of urethral recurrences.

## INTRODUCTION

Radical cystectomy (RC) with urinary diversion is a crucial urological procedure for the treatment of muscle-invasive bladder cancer (BCa) [1–3]. Furthermore, RC is an important therapeutic option for patients diagnosed with high-risk non–muscle-invasive BCa, recurrent non–muscle-invasive BCa, recurrent carcinoma *in situ* (CIS) following intravesical perfusion with bacillus Calmette-Guerin (BCG), or superficial carcinomas refractory to transurethral resection (TUR) and intravesical therapy [4].

BCa is the second-most common genitourinary malignancy, with the ninth-highest incidence among all cancers worldwide. This cancer is associated with very high expenses and high recurrence rates in general [5–7]. As the predominant pathological type of BCa, transitional cell carcinoma (TCC) is described as a pan-urothelial disease characterized by recurrent and multifocal metachronous tumors. These tumors arise from the intraluminal planting and transformation of malignant epithelial cells from urinary organs such as the renal pelvis, ureters, bladder, and urethra [8]. Therefore, the residual urethra is a common recurrence site after RC with urinary diversion, except in the upper urinary tract. Additionally, associations between urethral recurrence (UR) and other risk factors have been reported in previous investigations. Associated risk factors include pathological type, pathological staging, urinary diversion, histological grading, lymph node stage, multifocal BCa, prostatic involvement, and urethral margins. However, the predictive value of each factor remains incomplete and controversial. Meanwhile, no systematic schedule or complete follow-up instructions have been established to date. International guidelines also have overlooked these issues [8]. Therefore, a better understanding of the carcinogenic potential associated with various risk factors for UR after RC with urinary diversion is indispensable to evaluate the risks and guide follow-up strategies. Considering the lack of quantitative risk factor evaluation for UR after RC, we conducted the present meta-analysis to evaluate the predictive values of different risk factors to offer approximate guidance for prevention, timely diagnosis, and treatment of UR after RC with urinary diversion.

## RESULTS

### Search results

**Figure 1:**
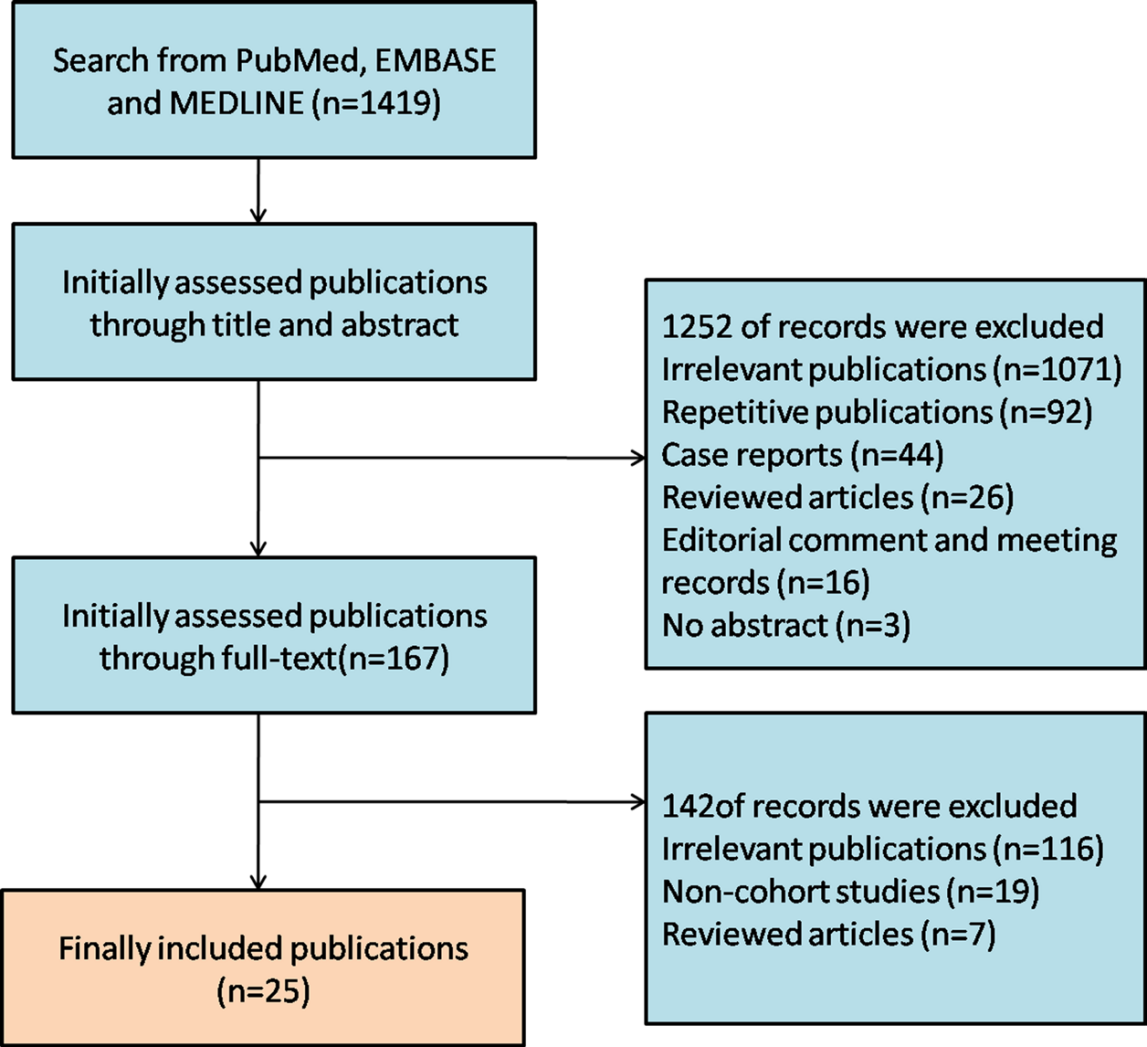
A flow diagram of the study selection The flow diagram showed the literature search for the relevant studies of the risk factors, follow-up and treatments of urethral recurrence following radical cystectomy and urinary diversion for bladder cancer.

Overall, 1419 studies were identified, 1252 of which were excluded after initial screening. Based on the selection procedures and the criteria described above, we reviewed the full text of the remaining 195 studies. Ultimately, 25 publications which included 9498 patients were considered eligible for inclusion in this meta-analysis (Figure 1).

### Database analysis

The characteristics of the 25 included studies are summarized in Supplementary Table 1. Of all 25 publications, 3 were published in the 1990s, 12 were published in the 2000s, and the remaining 10 were published after 2010, including 3 which were published in 2015 and 2016. There were 10 studies from the United States, 3 from Japan, 2 each from Spain and Switzerland, with one study each provided by Austria, Egypt, Germany, Finland, Korea, Canada, and Turkey.

### Patient characteristics

Among the 25 studies, 23 reported the gender and 20 provided the age of participants. There were 5520 male and 1218 female subjects, comprising an approximate 9:2 ratio. The average age was 62.3 years (range: 27 to 89). Follow-up duration was described in 17 studies, with a mean of 50.5 months (range: 1 to 254).

### Overall analysis of outcomes

A total of 362 cases (3.81%) of UR after RC with urinary diversion were reported among all 9498 patients. The recurrent cases were characterized by individual or multiple risk factors. The UR rates were also closely associated with the different risk factors. In addition to the mechanisms underlying the risk factors, heterogeneity, and publication bias also had to be considered, although Begg’s test, Egger’s test, and sensitivity analyses were performed.

### Pathological staging

**Figure 2:**
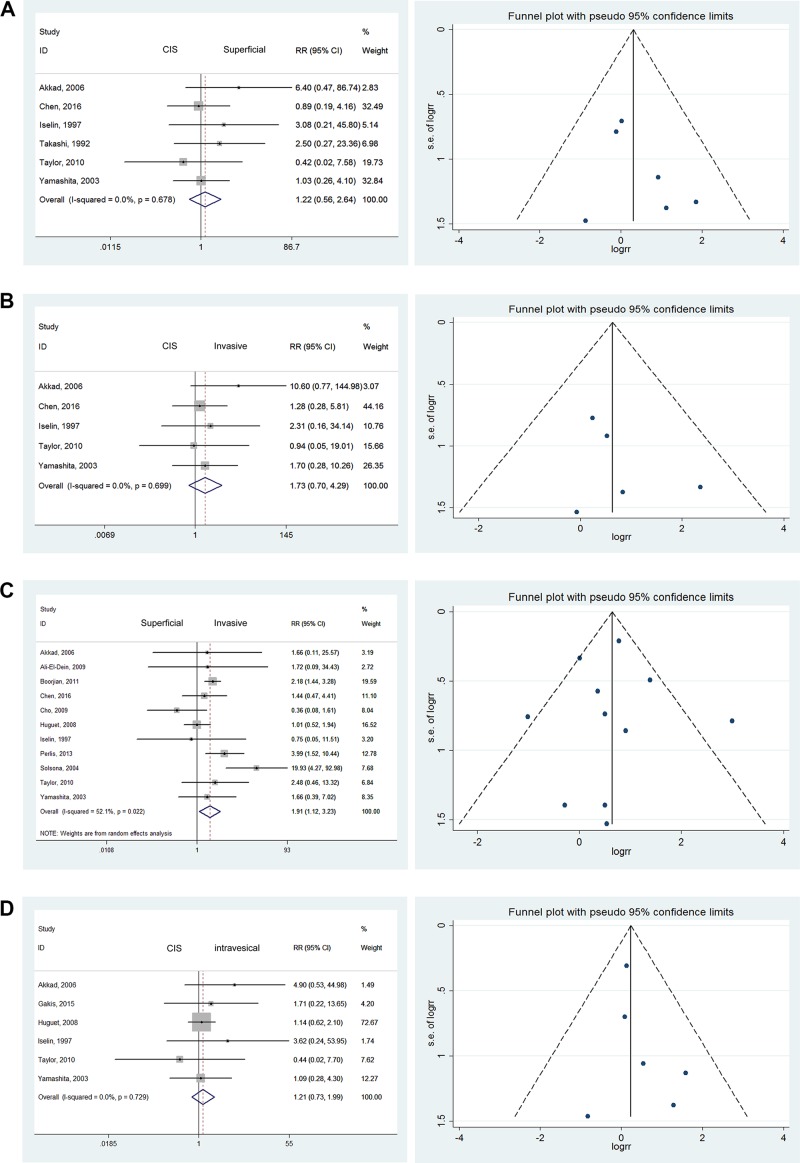

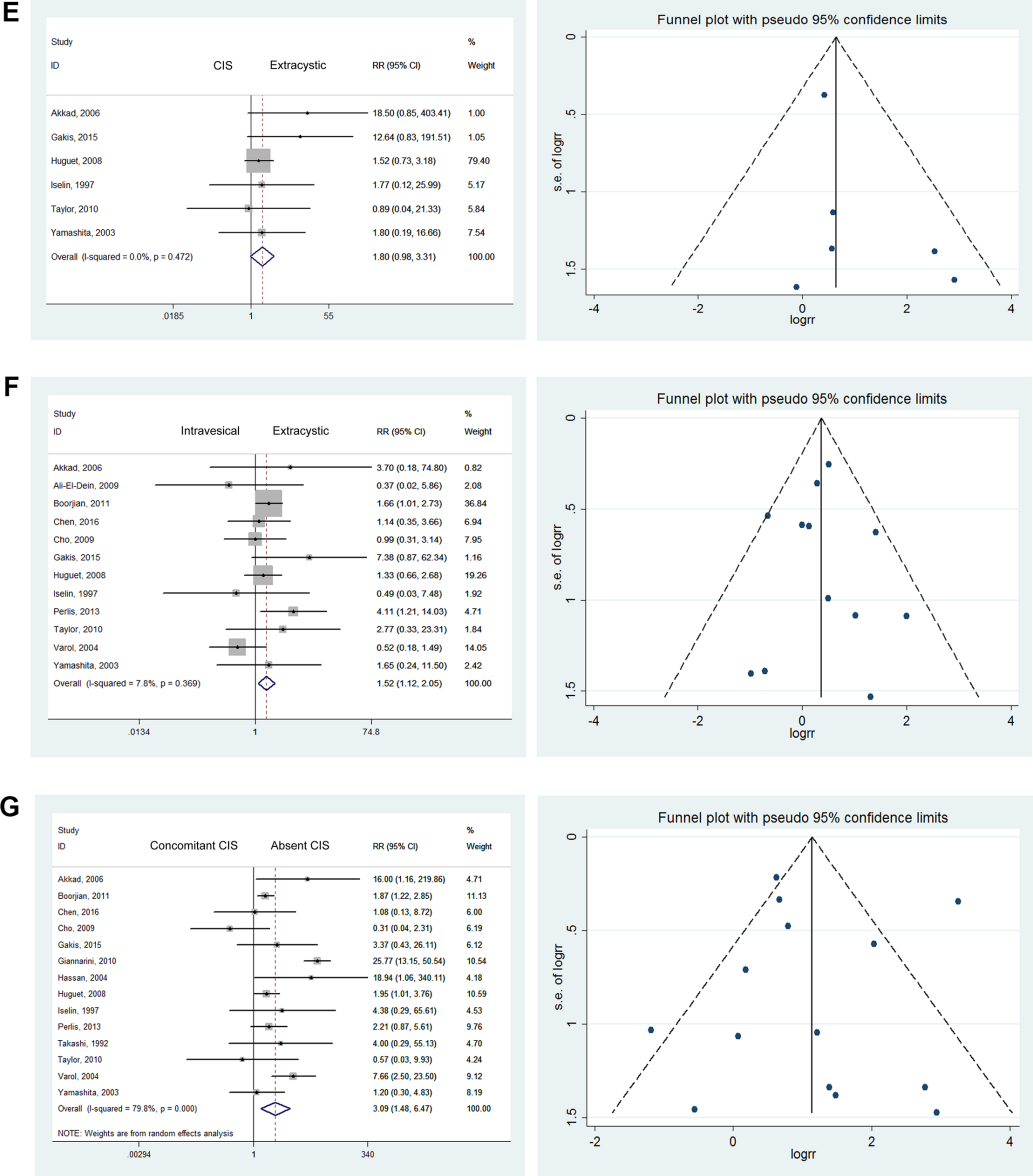
Forest plots and funnel plots of risk ratio for pathological stages The squares indicated the risk ratio (RR), and the horizontal lines indicated the 95% confidence interval (CI) for each included trial; the statistical weight of a trial in the meta-analysis was proportional to the size of each square; diamonds indicate the pooled risk ratio and 95% confidence interval, with the center indicating the point estimate and the left and the right ends indicating the 95% CI.

No statistically significant differences were noted when comparing CIS (Tis) with superficial carcinoma (RR = 1.22; 95% CI: [0.56, 2.64]). Similar results were found when comparing CIS versus invasive tumors (RR = 1.73; 95% CI: [0.70, 4.29]), CIS versus intravesical tumors (RR = 1.21; 95% CI: [0.73, 1.99]), and CIS versus extracystic tumors (RR = 1.80; 95% CI: [0.98, 3.31]). No heterogeneity was found among these studies [9–16]. Although insignificant outcomes were tested, CIS was always the greatest common risk factor observed. When comparing UR, statistically significant differences were noted between superficial and invasive tumors (RR = 1.91; 95% CI: [1.12, 3.23]) and between intravesical and extracystic tumors (RR = 1.52; 95% CI: [1.12, 2.05]) [9–15, 17–22]. We found mild heterogeneity in the latter group and moderate heterogeneity in the former (*I^2^* = 52.1%). Therefore, we chose a randomized model to evaluate the risk ratio of superficial versus invasive cancers. Others were tested using a fixed effects model (Figure 2A–2F).

### Effects of concomitant CIS

CIS bladder cancer, considered a significant risk factor for recurrence, is not usually a single lesion. It commonly occurs in various pathological stages and forms such as malignancies involving the urethral margin. Multifocality, involvement of the prostatic urethra, and response to BCG are closely associated with prognosis [23]. A significant difference found was that patients with concomitant bladder cancer CIS were more likely to suffer from UR than those without CIS (RR = 3.09; 95% CI: [1.48, 6.47]) [9, 10, 12–19, 21, 24, 25]. However, severe heterogeneity was noted among these results, with *I^2^* = 79.8%. Therefore, we used a randomized model to evaluate RR. The increased effects on UR were mainly due to the high-grade malignancies that were difficult treat radically (Figure 2G).

### Histological grading of bladder tumors

**Figure 3:**
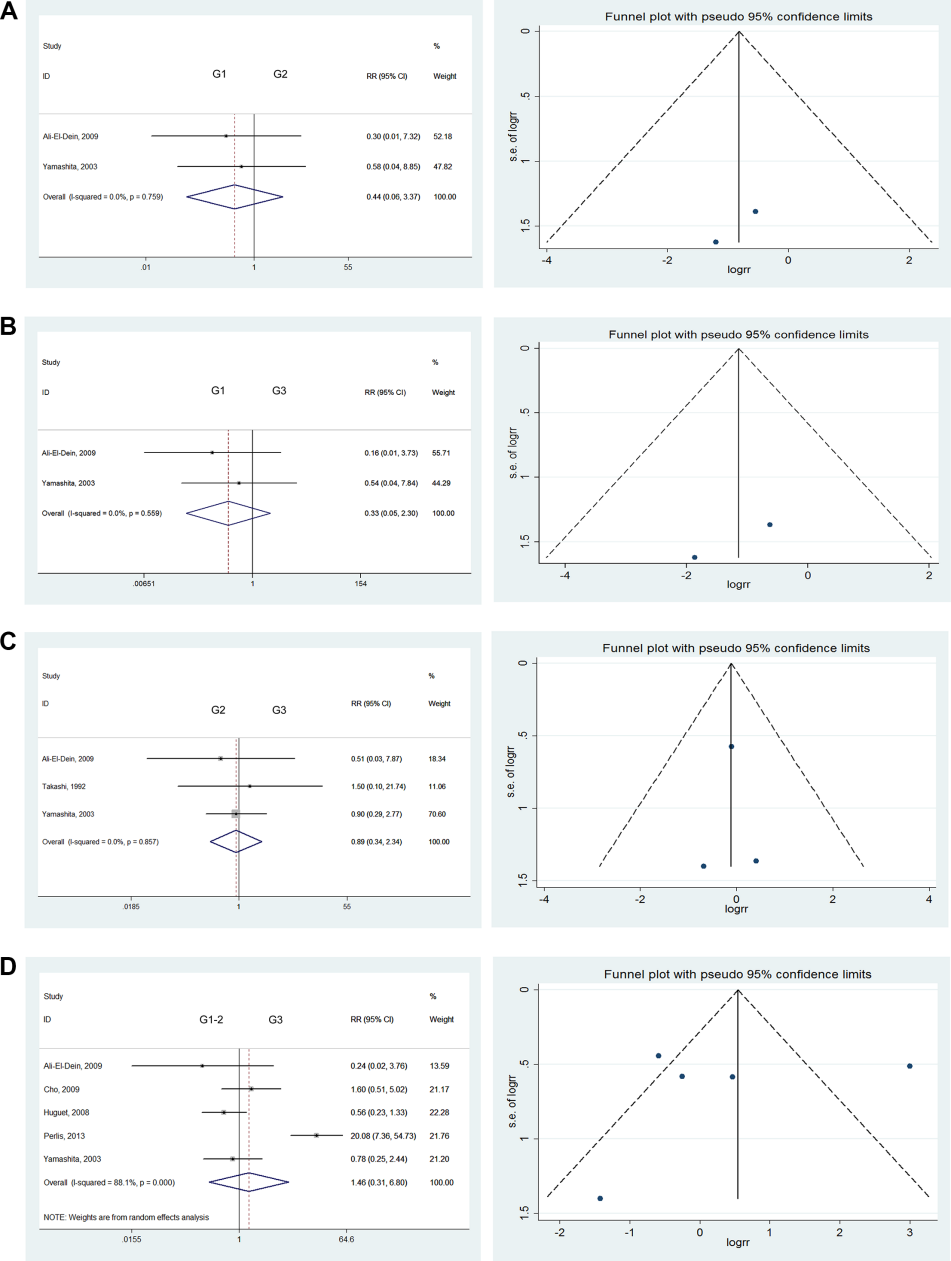

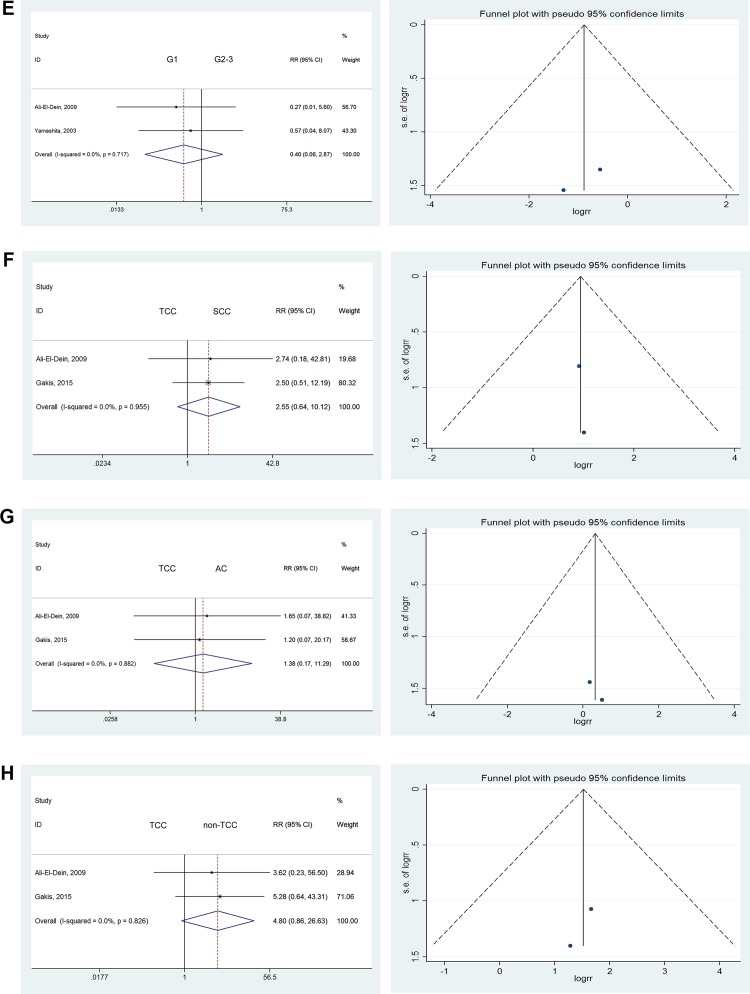
Forest plots and funnel plots of risk ratio for pathological type and clinical grading The squares indicated the risk ratio (RR), and the horizontal lines indicated the 95% confidence interval (CI) for each included trial; the statistical weight of a trial in the meta-analysis was proportional to the size of each square; diamonds indicate the pooled risk ratio and 95% confidence interval, with the center indicating the point estimate and the left and the right ends indicating the 95% CI.

There were no significant differences in the comparison of G1 versus G2 tumors (RR = 0.44; 95% CI: [0.06, 3.37]), G1 versus G3 tumors (RR = 0.33; 95% CI: [0.05, 2.30]), or G2 versus G3 tumors (RR = 0.89; 95% CI: [0.34, 2.34]). We also found no significant differences when comparing G1–2 versus G3 (RR = 1.46; 95% CI: [0.31, 6.80]) or G1 versus G2–3 tumors (RR = 0.40; 95% CI: [0.06, 2.87]) [12, 16, 17, 19, 20, 26]. Severe heterogeneity was found in the comparison of G1–2 versus G3 tumors (*I^2^* = 88.1%). Therefore, we used the randomized model to analyze these tumors. As no heterogeneity was found for all other comparisons, they were analyzed using the fixed effects model (Figure 3F–3H).

### Pathological types

According to the 2017 EAU guidelines, BCa pathological types were divided into TCC (more than 90% of cases), small-cell carcinomas (SCC), or adenocarcinoma (AC), among others. Given the higher prevalence of TCC versus other pathological types, we considered the UR rates associated with various pathological types. No significant differences were observed in comparisons of TCC versus other pathological types (Figure 3F–3H), such as TCC versus SCC (RR = 2.55; 95% CI: [0.64, 10.12]), TCC versus AC (RR = 1.38; 95% CI: [0.17, 11.29]), or TCC versus non-TCC (RR = 4.80; 95% CI: [0.86, 26.63]) [10, 20].

### Diversion types

**Figure 4:**
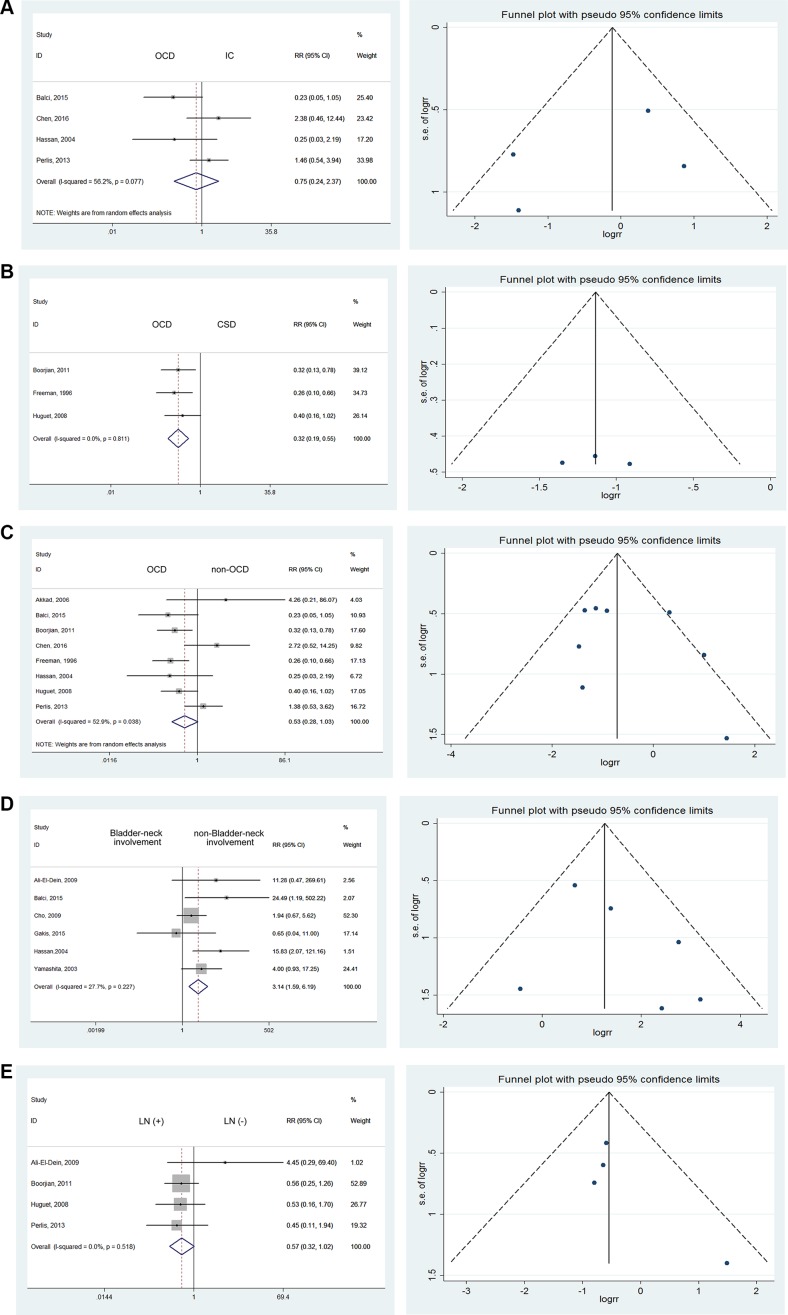

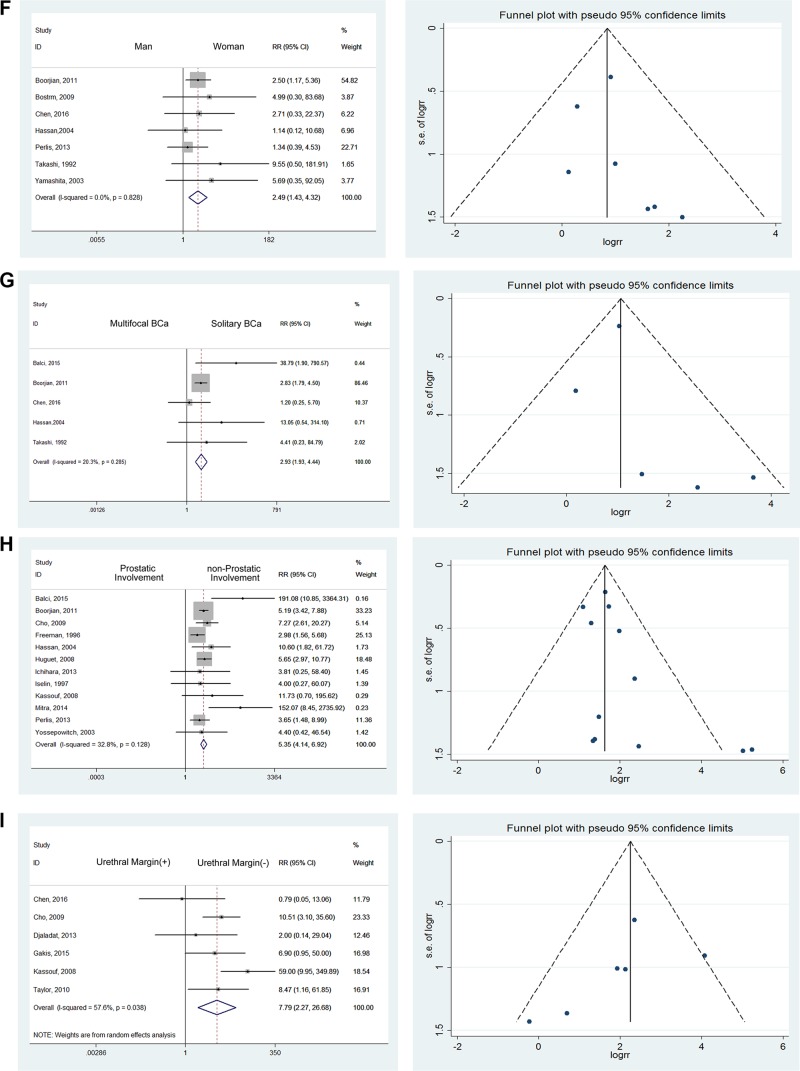
Forest plots and funnel plots of risk ratio for diversion types, bladder neck involvement, node involvement, gender influence, multifocal and solitary bladder cancer, prostate involvement, urethral margin The squares indicated the risk ratio (RR), and the horizontal lines indicated the 95% confidence interval (CI) for each included trial; the statistical weight of a trial in the meta-analysis was proportional to the size of each square; diamonds indicate the pooled risk ratio and 95% confidence interval, with the center indicating the point estimate and the left and the right ends indicating the 95% CI.

Given the lack of statistics about the relative risk of UR after three types of urinary diversion, we estimated the influence of these diversions on UR rates. A statistically significant difference was observed between orthotopic diversion (OCD) and cutaneous diversion (CSD). Specifically, the UR rate in OCD was 0.32 times that of CSD (RR = 0.32; 95% CI: [0.19, 0.55]). However, no statistically significant differences were noted in comparisons of OCD versus ileal conduit (IC) (RR = 0.75; 95% CI: [0.24, 2.37]) or OCD versus non-OCD (RR = 0.53; 95% CI: [0.28, 1.03]) [9, 12, 13, 17, 18, 25, 27, 28] (Figure 4A–4C).

### Bladder neck involvement

Among the 6 publications which presented comparisons of UR, we discovered a RR of 3.14 for bladder neck involvement (95% CI: [1.59, 6.19]) using the fixed effects model with mild heterogeneity (*I^2^* = 27.7%) [10, 14, 19, 20, 25, 27]. It can be easily inferred that bladder-neck involvement, an obvious risk factor, could confer a 3.14 times increased risk of UR, even though the difference was not statistically significant (Figure 4D).

### Node involvement

We investigated the influence of lymph node involvement on UR by comparing lymph node-positive with lymph node-negative cases. However, no significant difference was noted (RR = 0.57; 95% CI: [0.32, 1.02]), and no heterogeneity was detected using the fixed effects model [12, 17, 18, 20] (Figure 4E).

### Gender influence

In consideration of gender differences in the incidence of primary and secondary urethral carcinoma, we investigated the variations of UR accordingly. Among the 7 studies reporting gender-related data [9, 14, 16–18, 25, 29], we discovered that men were at higher risk for UR, with a RR of 2.49. No heterogeneity was detected using the fixed effects model (RR = 2.49; 95% CI: [1.43, 4.32]) (Figure 4F).

### Multifocal and solitary bladder cancer

Based on studies reporting that patients with multifocal BCa were at high risk of recurrence [9, 16, 18, 25, 27], we found a significant difference between solitary BCa and multifocal BCa (RR = 2.93; 95% CI: [1.93, 4.44]) with mild heterogeneity (I^2^ = 20.3%). (Figure 4G) Multiple lesions constituted a risk factor mainly through additional possibilities for dissemination and metastasis. The residual lesions were also refractory to the original treatments, resulting in epibiotic and occult lesions which could contribute to UR.

### Prostate involvement

Taking 12 studies into consideration [12, 15, 17, 18, 25–32], we discovered that prostate involvement was significantly associated with higher UR rates after RC with urinary diversion (RR = 5.35; 95% CI: [4.14, 6.92]). (Figure 4H) Mild heterogeneity was detected, with *I^2^* = 32.8%.

### Urethral margins

Taking 6 studies into consideration [9–11, 19, 32, 33], a significant difference was noted when comparing urethral margins. Patients with positive urethral margins were 7.79 times more likely to have UR (RR = 7.79; 95% CI: [2.27, 26.68]). (Figure 4I) Compared to prostatic involvement, the reasons for the high risk of UR were directly related to the malignant urethral remnants.

### Predicted values of follow-up protocol

Nineteen of the included studies described follow-up protocols and methods, including physical examination, radiologic imaging (X-rays, CT, and MRI), abdominal/transvaginal ultrasound, urethral cytology, cytology of urethral washings, cystourethroscopy, urethral dynamics, and bone scanning/scintigraphy. The remaining six publications did not mention follow-up methods. The distribution of follow-up methods is shown in Table 1.

**Table 1:** Table of diagnostic mode

Study, year	Symptomatic Diagnosis							Follow-up Diagnosis								
	N.	UD	Pain	P.M.	O.LUTs	U.I.		N.	P.E.	R.I.	Ultrasound	U.C.	U.W.C.	Cus.	U.D.	B.S.
Akkad, 2006	1	1	0	0	0	0		1	√	√	√	1	×	×	×	×
Balcı, 2015	3	n.r.	n.r.	n.r.	n.r.	n.r.		8	√	√	√	8	×	×	×	×
Boorjian, 2011	38	31	12	n.r.	n.r.	n.r.		47	√	√	√	√	√	×	×	×
Bostrm, 2009	8	n.r.	n.r.	n.r.	n.r.	n.r.		2	√	√	√	2	√	×	×	×
Cho, 2009	n.r.	n.r.	n.r.	n.r.	n.r.	n.r.		n.r.	×	√	×	×	√	×	×	×
Freeman, 1996	n.r.	n.r.	n.r.	n.r.	n.r.	n.r.		n.r.	√	×	×	√	√	×	×	×
Gaitonde, 2002	0	0	0	0	0	0		6	n.r.	n.r.	n.r.	n.r.	6	n.r.	n.r.	n.r.
Gakis, 2015	n.r.	n.r.	n.r.	n.r.	n.r.	n.r.		n.r	√	√	×	√	√	×	×	√
Giannarini, 2010	3	n.r.	n.r.	n.r.	n.r.	n.r.		21	√	√	√	√	√	×	√	√
Hassan, 2004	1	1	n.r.	n.r.	n.r.	n.r.		n.r.	√	√	×	√	×	×	×	×
Huguet, 2008	21	17	n.r.	2	3	1		13	√	√	√	13	√	×	×	×
Ichihara, 2013	n.r.	n.r.	n.r.	n.r.	n.r.	n.r.		n.r.	√	√	×	×	×	×	×	×
Iselin, 1997	n.r.	n.r.	n.r.	n.r.	n.r.	n.r.		n.r.	√	√	×	√	×	×	×	×
Kassouf, 2008	n.r.	n.r.	n.r.	n.r.	n.r.	n.r.		n.r.	√	√	×	√	×	×	×	×
Perlis, 2013	n.r.	n.r.	n.r.	n.r.	n.r.	n.r.		n.r.	×	√	×	√	√	×	×	×
Solsona, 2004	n.r.	n.r.	n.r.	n.r.	n.r.	n.r.		n.r.	√	√	×	√	×	×	×	×
Taylor, 2010	3	3	n.r.	n.r.	n.r.	n.r.		n.r.	n.r.	n.r.	n.r.	n.r.	n.r.	n.r.	n.r.	n.r.
Varol, 2004	1	1	0	0	0	0		14	n.r.	n.r.	n.r.	n.r.	14	n.r.	n.r.	n.r.
Yamashita, 2003	3	3	0	0	0	0		7	1	×	×	√	×	6	×	×
Yossepowitch, 2003	n.r.	n.r.	n.r.	n.r.	n.r.	n.r.		n.r.	√	√	×	√	√	×	×	×

B.S. = bone scan; Cus. = cystourethroscopy; N = numbers of patients; n.r. = no reported; O.LUTS = obstructive lower urinary tract symptoms; P.E. = physical examination; P.M. = penile mass; R.I. = radiologic imaging; U.C. = urethral cytology; UD = urethral discharge (urethral bleeding/purulent); U.D. = urethral dynamic; U.I. = urinary incontinence; U.W.C. = urethral washings cytology; “√” = Yes; “×” = No.

Among all 25 studies, 9 reported that UR was detected during routine follow-up in 118 patients (60.2% of all recurrences). Among these recurrences, 36 were discovered by urethral cytology, 28 through cytology of urethral washings, 6 through cystourethroscopy, and 1 by physical examination.

According to 11 of the included studies, another 82 patients (40.8% of all recurrences) with UR first reported symptomatic abnormalities, mainly including 57 patients with urethral discharge (urethral bleeding/purulent), 12 reporting pain, 2 with penile masses, 3 with obstructive lower urinary tract symptoms (LUTS), and 1 with incontinence. All patients were definitively diagnosed by urethral cytology or other criteria.

### Treatments and outcomes of patients with UR

As shown in Table 2, in 14 publications reported the treatments of 272 UR cases. Among these cases, 190 of underwent urethrectomy, 52 underwent urethra-sparing treatments (chemotherapy/radiation/ BCG), 17 underwent transurethral resection (TUR), 2 underwent penectomy and 1 underwent emasculation. An additional 10 patients refused treatment because of multiple distant metastases or other unknown reasons.

**Table 2:** Table of treatments and outcomes

Study, year	Treatments							Outcomes				
	urethrectomy	TUR	penectomy	emasculation	U.S.T.	N.T.		alive	D.BCa	D.M.D.	D.O.D.	NS.DR.
Akkad, 2006	2	0	0	0	0	0		0	0	1	1	0
Balcı, 2015	2	2	0	0	7	0		n.r.	n.r.	n.r.	n.r.	n.r.
Boorjian, 2011	73	0	0	0	6	6		12	35	n.r.	38	0
Bostrm, 2009	9	0	0	0	1	0		n.r.	n.r.	2	n.r.	n.r.
Cho, 2009	10	0	2	0	8	1		6	n.r.	n.r.	n.r.	7
Gaitonde, 2002	5	n.r.	n.r.	n.r.	n.r.	n.r.		4	n.r.	n.r.	n.r.	2
Giannarini, 2010	1	n.r.	n.r.	n.r.	0	n.r.		1	n.r.	n.r.	n.r.	n.r.
Huguet, 2008	31	2	0	0	1	0		n.r.	n.r.	n.r.	n.r.	n.r.
Ichihara, 2013	2	0	0	0	0	0		0	0	0	0	2
Iselin, 1997	n.r.	n.r.	n.r.	n.r.	n.r.	n.r.		1	0	1	0	0
Mitra, 2014	49	0	0	0	5	1		n.r.	21	n.r.	n.r.	n.r.
Takashi, 1992	1	0	0	1	0	0		0	2	0	0	0
Taylor, 2010	3	1	0	0	2	0		5	0	0	0	1
Varol, 2004	3	0	0	0	12	2		5	7	0	3	0
Yamashita, 2003	0	10	0	0	10	0		5	n.r.	n.r.	3	2
Yossepowitch, 2003	n.r.	2	n.r.	n.r.	n.r.	n.r.		2	0	1	0	0

D.BCa = die of bladder cancer; D.M.D. = die of metastatic disease; D.O.D. = die of other diseases; n.r. = no reported; NS.DR. = no statement of death reason; N.T. = no treatment; TUR = transurethral resection; U.S.T. = urethra-sparing treatment (chemotherapy/radiation/BCG).

12 studies obtaining 180 cases described the outcomes after UR. Forty-one patients were alive at the end of follow-up, 65 patients died of UC or other types of BCa, 5 died of distant metastases, and 45 died of other unrelated diseases. The cause of death for the remaining 14 patients was not stated.

### Sensitivity analyses and publication bias

We performed sensitivity analyses to identify the potential sources of heterogeneity of RR. For the severe heterogeneity detected in the comparison of G1–2 versus G3, the pooled improvement changed when omitting single studies in turn. Moreover, when we removed one study [17], the heterogeneity changed from *I^2^* = 88.1% to *I^2^* = 0.0%, accompanied by a change from (RR = 1.46; 95% CI: [0.31, 6.80]) to (RR = 0.77; 95% CI: [0.43, 1.37]). Although the heterogeneity was attributable to this study, we chose to retain it. Accordingly, we selected a random model to estimate this pooled outcome.

Although statistical tests suggested that there was no evidence of publication bias using both Begg’s test and Egger’s test (all *P* values were greater than 0.05), it remained difficult to rule out publication bias through visual inspection of the funnel plot.

## DISCUSSION

Recurrences in the residual urethra after RC with urinary diversion are intractable and mainly occur in the urethra below the bladder. In addition to exploring the treatments for recurrent carcinoma, estimating UR risks and taking precautions are also crucial. Risk factors reported by previous studies have been proven to affect UR rates. Nonetheless, the relative risks are still needed for appropriate evaluation of these factors. Additionally, effective follow-up protocols should be developed and implemented to effectively detect recurrences.

In general, pathology staging, a critical prognostic factor, has been significantly associated with local and systemic recurrence rates. This association is found to be similarly significant for UR rates [27, 34, 35]. We found that higher UR rates occurred in lower-stage malignancies such as superficial and intravesical tumors compared with invasive and extracystic tumors. This finding may be ascribed to the following reasons: (1) The initial early-stage tumors, inadequately controlled by BCG or simple TUR, progressed to UR. (2) Follow-up and urinary symptom surveillance for patients with early-stage tumors was less stringent than for advanced tumors. (3) Early-stage tumors are associated with longer follow-up and survival. Accordingly, more patients may have been diagnosed with secondary urethral lesions over time.

Studies have demonstrated that urinary diversion methods are closely related to UR rates [11, 25, 36]. Many publications have reported higher UR rates associated with OCD compared with other diversion types such as IC and CSD [18, 27]. However, Djaladat et al reported that UR rates in patients with OCD were significantly lower than those with CSD (2.9% versus 11.1%, respectively) [33]. Balci et al also reported that UR rates in patients with OCD and IC were 1.4% and 6.2%, respectively [27].

Based on the present meta-analysis, we discovered that OCD was a relatively protective factor for UR compared with the other 2 types of urinary diversion. Possible explanations are as follows: (1) OCD may contribute to early diagnosis to prevent UR, due to urination through the urethra. (2) OCD provided a protective effect from urine compared to juxtaposition of the ileum [33]. (3) OCD can be performed more safely than other urinary diversions [37]. (4) OCD may avoid the immune responses to carcinogenic antigens caused by urothelial and intestinal tissues [38]. (5) Another reason might be selection bias because patients with other risk factors such as advanced staging, prostatic involvement, and multifocal lesions may be more likely to undergo non-orthotopic diversion.

Bladder neck involvement, a significant prognostic factor in bladder carcinomas, has been implicated in a high proportion of urothelial recurrences after RC with urinary diversion for women with bladder cancer [39, 40]. Ali-El-Dein reported that bladder neck tumors were usually accompanied by high-grade and stage as well as node-positive disease [20]. Concomitant involvement of the distal urethra at the time of RC was observed more frequently among women with bladder neck tumors [10, 40]. Therefore, bladder neck involvement was not considered to be an individual risk factor increasing the rate of UR after RC. Rather, it was a concomitant finding closely associated with other risk factors. Accordingly, it appears rational to advocate preoperative cystoscopy to assess bladder-neck involvement and guide operative procedure selection and follow-up.

According to the meta-analysis comparing gender-specific UR rates, we discovered that males were relatively prone recurrence after RC. Possible reasons are as follows: (1) The relatively complex male physiological structure, including the longer urethra and relatively narrow and intricate anatomic structures may make implantation metastasis more likely. (2) Prostate invasion, with its high associated risk of UR, is unique to men. (3) Prophylactic urethrectomy was more common among women, especially those without OCD. (4) Given the population bias in these studies, we considered the differences in the population of men with BCa. These characteristics mainly contributed to the high UR rates among men.

Prostate involvement was classified as superficial or invasive. Some studies demonstrated that UR rates were as high as 30–37% in patients with prostate involvement [41, 42]. Stein and his colleagues also reported that UR rates in patients with invasive prostate involvement were higher than in those with superficial involvement. They also reported that rates in both groups were significantly higher than among patients without prostate involvement [43]. Cho et al also reported that the estimated risk of prostatic urethral involvement and stromal invasion was 6 to 8 times higher, respectively, considering each as independent risk factors [19]. Unfortunately, there no studies have investigated the risk ratios of prostate involvement for UR. Therefore, we calculated the predictive values in the present meta-analysis.

Finally, we found BCa patients with prostate involvement were at higher risk of recurrence. We considered the high risk associated with prostate involvement for the following reasons: (1) Malignant potential was higher for bladder cancer with prostate involvement. (2) As part of the urethra, an involved prostate may transfer malignancy to urethra more easily. (3) Incomplete prostate resection during RC may contribute to subsequent UR from the remnant. Given the high risk of prostate involvement, a preoperative prostatic urethral biopsy or frozen section analysis should be conducted to guide the operative methods and follow-up.

Intraoperative frozen section analysis at the time of cystectomy before urinary tract diversion has long been regarded as a standard protocol to guide the excision of residual urethral tissues to obtain negative urethral margins and decrease UR rates [8]. Nevertheless, the predictive values and limitations have not been explicitly analyzed. Many studies have demonstrated that patients with positive urethral margins during intraoperative frozen section or final pathological evaluation were at higher risk of UR. By considering these studies in this meta-analysis, we found positive urethral margins was one of the significant risk factors for UR.

Although TRU biopsy of the prostatic urethra facilitates assessment of urethral margins, some studies have demonstrated that a positive TUR biopsy of the prostatic urethra does not reliably predict a positive final urethral margin [11]. In consequence, except for evaluation of prostate involvement, assessment of the urethral margin was also essential during RC to guide treatment and follow-up.

According to review of the studies included in this meta-analysis, urethral cytology was found to be a convenient and non-invasive method to predict UR. However, deciduous and necrotic intestinal epithelial cells in the urine flowing through the urinary diversion may obscure or be confused with urethral cancer cells. Additionally, the time that urine is stored in the neobladder or continent reservoir may also lead to anaplasia of urothelium cells. These confounding factors can result in a certain degree of inaccuracy of urethral cytology.

We also discovered a longer median time to recurrence in symptomatic versus asymptomatic patients. Meanwhile, cancer-specific survival (CSS) and overall survival (OS) were significantly higher in asymptomatic than symptomatic patients [7, 24]. Balci et al also reported that symptomatic recurrence was associated with more frequent metastases and worse prognoses compared to patients with asymptomatic recurrence [27]. UR diagnosed by routine follow-up was likely associated with higher survival rates because most invasive URs cause symptoms. However, most URs identified by follow-up were superficial. Therefore, routine or extended follow-up should be performed to facilitate early detection of patients at high risk of UR. Additionally, follow-up protocols based on a risk-adapted strategy could avoid excessive testing for patients at lower risk of recurrence [44].

In addition to risk factors and follow-up protocols, treatments after UR were also strongly associated with outcomes. Generally, treatment methods are based on the pathologic staging of recurrent urethral tumors. Urethrectomy is the most common method for treatment of UR, occasionally accompanied by chemotherapy or radiation therapies for invasive tumors [27]. Prophylactic urethrectomy was also performed in particular circumstances. Given the convenience and reduced time requirement, concurrent urethrectomy during RC was an appropriate option for women without CSD to reduce the risk of UR. Nevertheless, for men, no evidence has proven that immediate urethrectomy contributed to a survival benefit compared with delayed urethrectomy [1, 24]. Other than surgical treatment, BCG administration is considered for recrudescent urethral tumors diagnosed at early pathological stages. For example, intraurethral BCG treatment has been used as monotherapy for CIS discovered in single or small numbers in the remnant urethra. Meanwhile, intracavitary BCG administration combined with resection was commonly used for patients with UR at the Ta or low-grade stages.

In brief, different treatments after recurrence were closely related to outcomes for patients with UR. However, no publications have reported significant connections with survival after UR with urethrectomy. Predictions of risk could provide direction for preventive urethrectomy. Accordingly, research exploring survival and its relationship with treatments after UR following RC with urinary diversion would be worthwhile.

## CONCLUSIONS

In the present meta-analysis, we clearly analyzed and weighed various risk factors. Ultimately, pathological staging (CIS, superficial, and intravesical BCa), urinary diversions (non-OCD), gender (male), prostatic involvement, positive urethral margins, bladder neck involvement, and multifocal BCa conferred statistically significant elevated risks of UR. Consequently, precautions and more strict follow-up protocols should be implemented for BCa patients with these risk factors after RC and urinary diversion, such as the urethral cytology, urethral washings cytology and radiologic imaging. Additionally, outcomes of UR after RC for patients with BCa were worse due to late diagnosis. Patients with symptomatic recurrence had significantly worse prognoses was than those diagnosed through regular follow-up. Therefore, early diagnosis plays a critical role in the prognosis of UR. Follow-up methods were also crucial for diminishing the likelihood of UR. As a simple and noninvasive method, urethral cytology was the most common way to diagnose UR. Urethrectomy, either simple or combined with chemotherapy, radiation or BCG, was usually used to treat UR. Other therapies such as TUR, penectomy and emasculation were used in specific recurrences. Prophylactic urethrectomy should also be planned for patients at high risk of UR.

## MATERIALS AND METHODS

### Search strategy

The literature search was performed using PubMed, EMBASE, and MEDLINE. We searched for studies published between 1971 and 2016, limited to English, and filtered using combinations of the following MeSH key terms: “Carcinoma, Transitional Cell”, “Urinary Bladder Neoplasms”, “Urothelial Carcinoma of the Bladder”, “Cystectomy”, “Urinary Diversion”, “Urologic Neoplasms”, and “Neoplasm Recurrence, Local”. Other publications were incorporated by scanning article references and including studies, reviews, meta-analyses, and guidelines.

### Study selection criteria

Inclusion criteria were: (1) English publications; (2) Retrospective or prospective cohort studies; (3) BCa patients who underwent RC and urinary diversion (4) Cases with UR, defined as urothelial recurrence below the bladder; (5) Studies which described the relevance of UR and risk factors; (6) Outcomes were presented in the form of sample sizes. When publications from the same institution and study were obtained, we used the one with more patients and the most credible information. We excluded case reports, case control studies, meeting abstracts, conference proceedings, repeated publications, and publications which included RC patients with urogenital neoplasms other than bladder cancer and bladder cancer patients who underwent radiotherapy or neoadjuvant chemotherapy.

### Selection procedures and data extraction

Initially, all studies were screened by the following process: (1) All gathered studies were screened via titles and abstracts, and (2) Full-texts of selected articles was reviewed to identify satisfactory studies. After these steps, the following information was independently extracted from the chosen studies by two authors: first author, year of publication, study design, sample size, gender, age, follow-up duration, pathological types, diversion types, pathological stages, clinical grades, diagnosis mode (symptomatic or asymptomatic), and other risk factors such as multifocal bladder cancer, bladder neck involvement, prostate invasion, positive lymph nodes (LN), positive urethral margin, and carcinoma *in situ* (CIS).

### Assessment of study quality

Study quality was assessed using the Newcastle-Ottawa Scale (NOS) of cohort studies, which includes 8 aspects. All studies included in this meta-analysis were identified as high quality. The median score and range of all selected studies was 8 (7–9). This meta-analysis was performed according to the Preferred Reporting Items for Systematic Reviews and Meta-Analyses (PRISMA) statement.

### Statistical analyses

Since our data were all dichotomous, we used risk ratios to evaluate the several risk factors of UR after RC with urinary diversion for bladder cancer. The pooled risk ratios were estimated using STATA software (Version 12.0). A *P*-value less than 0.05 for any test or model was considered to be statistically significant. We used Cochran’s χ^2^-based *Q* test and the I-squared test to assess inter-study heterogeneity. If there was no significant heterogeneity (*P* > 0.10 or I^2^ < 50%), then the pooled outcome was evaluated using the fixed effects model (Mantel-Haenszel). The random effects model (DerSimonian and Laird) was used when significant heterogeneity was found. Additionally, a sensitivity analysis was conducted to determine the influence of individual trials on the overall pooled results.

Possible publication bias was considered using the Begg’s rank correlation test and Egger’s linear regression test. Additionally, funnel plots were employed to assess potential publication bias.

## SUPPLEMENTARY MATERIALS TABLE





## Abbreviations

RC: radical cystectomy
BCa: bladder cancer
CIS: carcinoma *in situ*
BCG: bacillus Calmette-Guerin
TUR: transurethral resection
TCC: transitional cell carcinoma
UR: urethral recurrence
SCC: small-cell carcinomas
AC: adenocarcinoma
OCD: orthotopic diversion
IC: ileal conduit
CSD: cutaneous diversion
LUTS: lower urinary tract symptoms
CSS: cancer-specific survival
OS: overall survival.
