# Supplementary material for: Risk factors, follow-up, and treatment of urethral recurrence following radical cystectomy and urinary diversion for bladder cancer: a meta-analysis of 9498 patients
Source: Oncotarget. 2017 Dec 19;9(2):2782–96. doi: 10.18632/oncotarget.23451 (PMC5788679; doi:10.18632/oncotarget.23451)
Supplement: Supplementary file 1 [file oncotarget-09-2782-s001.pdf]

Risk factors, follow-up, and treatment of urethral recurrence following radical cystectomy and urinary diversion for bladder cancer: a meta-analysis of 9498 Patients

SUPPLEMENTARY MATERIALS

Supplementary Table 1: Basic information of included literatures. See Supplementary_Table_1
