# Supplementary material for: Risk factors, follow-up, and treatment of urethral recurrence following radical cystectomy and urinary diversion for bladder cancer: a meta-analysis of 9498 patients
Source: Oncotarget. 2017 Dec 19;9(2):2782–96. doi: 10.18632/oncotarget.23451 (PMC5788679; doi:10.18632/oncotarget.23451)
Supplement: Supplementary file 2 [file oncotarget-09-2782-s002.docx]

**Supplementary Table 1: Basic information of included literatures**

| Study  Author, year | Total sample size/N(pat.) with UR | Sex of UR patients  (m/w) | Median(or range)age  (years) | Median (or range)  follow-up time (months) | Median (or range)  Time to recurrence  (months) | Pathological types  (N,% pat. with same-type) | Diversion types  (N,% pat. with same-type) | Multifocal bladder Ca(N, % of pat. with same-risk) | Bladder-neck involvement  (N, % of pat. with same-risk) | Prostate invasion  (N, % of pat. with same-risk) | LN (+)  (N, % of pat. with same-risk) | Urethral margin(+)  (N, % of pat. with same-risk) | Concomitant CIS  (N, % of pat. with same-risk) | Pathological stages  (N, % of pat. with same-risk) | Clinical- grades  (N,% of same grade) |
| --- | --- | --- | --- | --- | --- | --- | --- | --- | --- | --- | --- | --- | --- | --- | --- |
| Akkad, 2005 | 85/2 | 0/2 | 64.5  (34-82) | 49.8(5-149) | 50.5 | TCC (2, 2.4) | OCD (2, 4.3) | 1, n.r. | n.r. | n.r. | n.r. | n.r. | 1, 20 | Tis (1, 20)  ≤T1 (1, 3.1)  T2 (1, 5.9) | n.r. |
| Ali-El-Dein, 2009 | 180/2 | 0/2 | 50.6 | 57 (5-137) | n.r. | TCC (1, 2.6)  SCC (1, 0.9) | n.r. | n.r. | 1, 3.6 | n.r. | 1, 3.0 | n.r. | 0, 0 | P3a (1, 1.4)  P3b (1, 2.2) | G2 (1, 1.4)  G3 (1, 2.9) |
| Balcı, 2015 | 287/11 | 11/0 | 54.9 | n.r. | 28.6 | UC (11,100) | OCD (2,1.4)  IC (9,6.2) | 2, 6.3 | 2, 4.2 | 4, 33.3 | n.r. | n.r. | n.r. | n.r. | n.r. |
| Boorjian, 2011 | 1506/85 | 78/7 | n.r. | 13.5 (10.5-18.4) | 13.3 (6.1-13.2) | n.r. | OCD (5, 2.1)  CSD (80, 6.4) | 61,8.6 | n.r. | 21, 21.8 | 6, 3.3 | n.r. | 51, 7.6 | ≤T1 (41, 9.1)  T2 (25, 4.4)  T3/4 (19, 3.9) | n.r. |
| Bostrm, 2009 | 248/10 | 10/0 | 64 (38-80) | 75 (1-250) | 18 (10-96) | n.r. | n.r. | n.r. | n.r. | n.r. | n.r. | n.r. | n.r. | n.r. | n.r. |
| Chen, 2016 | 111/6 | 5/1 | 67 (34-88) | 40.8 (3-155) | n.r. | TCC (6, 5.4) | OCD (4, 8.5)  IC (2, 3.6) | n.r. | n.r. | n.r. | n.r. | 0, 0 | 5, 5.7 | Tis (2, 11.1)  ≤T1 (5, 12.5)  T2 (2, 7.7)  T3/4 (4, 9.3) | n.r. |
| Cho, 2009 | 294/13 | 13/0 | 61 (27-89) | 54 (6-227) | 17 (6-63) | n.r. | n.r. | n.r. | 6, 6.7 | 6, 19.4 | n.r. | 2, 40 | 1, 1.6 | ≤T1 (2, 2.0)  T2 (7, 6.6)  T3/4 (4, 4.4) | G1-2 (4, 6.3)  G3 (9, 3.9) |
| Djaladat, 2013 | 33/2 | 2/0 | 71 (51-83) | 57.6 (1.2-252) | 28.8 | n.r. | n.r. | n.r. | n.r. | n.r. | n.r. | 1, 9.1 | n.r. | n.r. | n.r. |
| Freeman, 1996 | 436/34 | 34/0 | 64 | 72 (12, 252) | 19.2 (2.4-105.6) | TCC (34, 7.8) | OCD (5, 2.9)  CSD (29, 11.1) | 13, 17.33 | n.r. | n.r. | n.r. | n.r. | n.r. | n.r. | n.r. |
| Gaitonde, 2002 | 105/6 | n.r. | n.r. | n.r. | n.r. | n.r. | n.r. | n.r. | n.r. | n.r. | n.r. | n.r. | n.r. | n.r. | n.r. |
| Gakis, 2015 | 297/7 | 0/7 | 54 (47, 57) | 64 (25, 116) | 30 (8-64) | TCC (6, 4.0)  SCC (2, 1.6)  AC (0, 0) | n.r. | n.r. | 0, 0 | n.r. | n.r. | 1, 14.3 | 1, 7.1 | Tis (1, 7.1)  ≤T2 (5, 4.2)  ≥T3 (1, 0.6) | n.r. |
| Giannarini, 2010 | 479/21 | n.r. | 65.7 (58.4, 75.4) | 51.6 (3.6-250.8) | 74.4 (27.6, 194.4) | n.r. | n.r. | n.r. | n.r. | n.r. | n.r. | n.r. | 13, 61.9 | n.r. | n.r. |
| Hassan, 2004 | 196/1  194/4 | 3/1 | n.r. | n.r. | n.r. | TCC (5, 1.3) | OCD (1, 0.5)  IC (4, 2.1) | 1, 2.8 | 1, 25 | 3, 6.7 | n.r. | n.r. | 5, 3.5 | n.r. | n.r. |
| Huguet, 2008 | 729/34 | 34/0 | 64 (51-73) | 38 (8-121) | 13.9 (7-21) | TCC (34, 4.7) | OCD (5, 2.3)  CSD (29, 5.7) | n.r. | n.r. | 18, 14.9 | 3, 2.7 | n.r. | 17, 6.9 | ≤T1 (15, 5.5)  T2 (8, 7.2)  T3/4 (8,3.4) | G1-2 (6, 3.0)  G3 (28, 5.3) |
| Ichihara, 2013 | 101/2 | n.r. | 68 (44-83) | 44 (1.4-175) | n.r. | TCC (2, 2.0) | n.r. | n.r. | n.r. | 1, 4.8 | n.r. | n.r. | n.r. | n.r. | n.r. |
| Iselin, 1997 | 70/2 | 2/0 | 62 (36-83) | 35 | 10.5 | n.r. | OCD (2, 2.9) | n.r. | n.r. | 1, 7.1 | n.r. | n.r. | 1, 7.7 | Tis (1, 7.7)  ≤T1 (1, 2.5)  P3/4 (1, 4.3) | n.r. |
| Kassouf, 2008 | 252/2 | 2/0 | 61 (53-80) | 48 (4-161) | n.r. | n.r. | n.r. | n.r. | n.r. | 0, 0 | n.r. | 1, 100 | n.r. | n.r. | n.r. |
| Mitra, 2014 | 2029/55 | n.r. | n.r. | n.r. | n.r. | TCC (55, 2.7) | n.r. | n.r. | n.r. | 5, 3.5 | n.r. | n.r. | n.r. | n.r. | n.r. |
| Perlis, 2013 | 574/18 | 15/3 | 70 (32–88) | n.r. | 42 (8-56) | TCC (18, 3.1) | OCD (6, 3.9)  IC (10, 2.7) | n.r. | n.r. | 7, 13.2 | 2, 1.8 | n.r. | 11, 5.3 | ≤T1 (12, 7.1)  T2 (3, 2.7)  T3/4 (3, 1.3) | G1-2 (12, 23.1)  G3 (5, 1.1) |
| Solsona, 2004 | 318/9 | n.r. | n.r. | n.r. | n.r. | TCC (9, 2.8) | n.r. | n.r. | n.r. | n.r. | n.r. | n.r. | n.r. | ≤T1 (7, 15.2)  ≥T2 (2, 0.8) | n.r. |
| Takash, 1992 | 30/2 | 2/0 | 59.3 (36-75) | n.r. | n.r. | n.r. | n.r. | 2, 12.5 | n.r. | n.r. | n.r. | n.r. | 1, 16.7 | Tis (1, 16.7)  ≤T1 (2, 6.7) | G2 (1, 8.3)  G3 (1, 5.6) |
| Taylor, 2010 | 259/6 | n.r. | 60.8 (31.1–80.3) | 54 (0-187.2) | 28.8 (8.4- 43.2) | n.r. | n.r. | n.r. | n.r. | n.r. | n.r. | 1, 16.7 | 0, 0 | Tis (0, 0)  ≤T1 (4, 3.4)  T2 (1, 1.6)  T3/4 (1, 1.2) | n.r. |
| Varol, 2004 | 371/15 | n.r. | 63 (52-76) | n.r. | 14 (3-70) | n.r. | n.r. | n.r. | n.r. | n.r. | n.r. | n.r. | 11, 11.2 | ≤T2 (5, 2.7)  ≥T3 (10, 5.3) | n.r. |
| Yamashita, 2003 | 73/10 | 10/0 | 64(40-74) | 60.5 (2-254) | n.r. | n.r. | n.r. | n.r. | 8, 27.6 | n.r. | n.r. | n.r. | 2, 20 | Tis (2, 20)  ≤T1 (8, 19.5)  T2 (1, 12.5)  T3/4 (2, 11.8) | G1 (0, 0)  G2 (4, 20)  G3 (6, 22.2) |
| Yossepowitch, 2003 | 241/3 | n.r. | 58 (31-76) | 44 (1-153) | n.r. | n.r. | n.r. | n.r. | n.r. | 1, 4.8 | n.r. | n.r. | n.r. | n.r. | n.r. |

AC=adenocarcinoma; Ca.=cancer; CSD=cutaneous diversion; IC=ideal conduit; LN=lymph node; m=men; N=number; n.r.=not reported; OCD=orthotopic diversion; pat.=patients; SCC=squamous cell carcinoma; TCC=transitional cell carcinoma; UR=urethral recurrence; w=women.
